# Investigation of the activity of transposable elements and genes involved in their silencing in the newt *Cynops orientalis*, a species with a giant genome

**DOI:** 10.1038/s41598-021-94193-6

**Published:** 2021-07-20

**Authors:** Federica Carducci, Elisa Carotti, Marco Gerdol, Samuele Greco, Adriana Canapa, Marco Barucca, Maria Assunta Biscotti

**Affiliations:** 1 Dipartimento di Scienze della Vita e dell’Ambiente, Università Politecnica delle Marche, Via Brecce Bianche, 60131 Ancona, Italy; 2 Dipartimento di Scienze della Vita, Università degli Studi di Trieste, Via L. Giorgieri, 5, 34127 Trieste, Italy

**Keywords:** Evolutionary genetics, Gene expression, Genome, Evolution, Genetics

## Abstract

Caudata is an order of amphibians with great variation in genome size, which can reach enormous dimensions in salamanders. In this work, we analysed the activity of transposable elements (TEs) in the transcriptomes obtained from female and male gonads of the Chinese fire-bellied newt, *Cynops orientalis*, a species with a genome about 12-fold larger than the human genome. We also compared these data with genomes of two basal sarcopterygians, coelacanth and lungfish. In the newt our findings highlighted a major impact of non-LTR retroelements and a greater total TE activity compared to the lungfish *Protopterus annectens*, an organism also characterized by a giant genome. This difference in TE activity might be due to the presence of young copies in newt in agreement also with the increase in the genome size, an event that occurred independently and later than lungfish. Moreover, the activity of 33 target genes encoding proteins involved in the TE host silencing mechanisms, such as *Ago/Piwi* and *NuRD* complex, was evaluated and compared between the three species analysed. These data revealed high transcriptional levels of the target genes in both newt and lungfish and confirmed the activity of NuRD complex genes in adults. Finally, phylogenetic analyses performed on *PRDM9* and *TRIM28* allowed increasing knowledge about the evolution of these two key genes of the NuRD complex silencing mechanism in vertebrates. Our results confirmed that the gigantism of the newt genomes may be attributed to the activity and accumulation of TEs.

## Introduction

Genome size varies considerably across eukaryotes and it is correlated neither with the number of genes, nor with the morpho-functional complexity of a species. Besides having a significant impact on the number and the size of introns^1,2^, as well as on the placement of regulatory regions, genome size is profoundly influenced by the relative abundance and activity of transposable elements (TEs)^3–5^. These genetic elements constitute a large fraction of the repetitive genomic DNA and are able to move throughout genomes through mechanisms of transposition based on a DNA intermediate molecule, in the case of DNA transposons, and on an RNA intermediate molecule, in the case of retrotransposons (LTR and non-LTR retroelements).

Adaptive and non-adaptive evolutionary hypotheses have been proposed to explain the surprising genome size variation observed in eukaryotes^6–8^. However, a scientific consensus about this issue is still far from being reached, as the genome size observed in extant metazoan species is the result of the combination between complex events that may have occurred ancestrally or relatively recently. These include auto- or allo-polyploidization events^9,10^, large-scale structural variations that may include deletions and insertions, expansions of tandem repeat arrays, and changes in the relative abundance of TE families^11^. Moreover, the amount of nuclear DNA does not only affect the size of both cells and nuclei in a given species, but also determines the duration of cell cycle and influences metabolic rates and the speed of development^12–14^.

The amphibian order Caudata is characterized by a great variation in genome size between metamorphic and neotenic species, which show the smallest and the largest genomes, respectively. As of February 2021, the 170 urodele species listed in the Animal Genome Size database display a genome size ranging from 13.89 to 120.60 Gb^15^. The highest values belong to salamanders, which together with lungfish are the record holders for the largest genomes among extant vertebrates.

Even though several factors, such as metabolic rate and cell cycle duration, have been associated with amphibian genome size^8,16,17^, the molecular mechanisms favouring the persistence of extreme repetitive DNA contents in salamander genomes remain to be addressed.

Evidence suggest that the gigantism of urodele genome is not attributable to polyploidy but rather to a reduced DNA loss rate and to the accumulation of TEs, in particular of LTR retrotransposons^18–20^. Among phylogenetically different crown salamanders, differences in genome composition have been linked to a balance between TE proliferation and host silencing mechanisms. The increase in genome size could be due to a reduced level of TE suppression, which can be likely traced back to the latest common ancestor of crown salamanders and was inherited by all the organisms belonging to this lineage. Moreover, an ongoing proliferation of *Ty3/Gypsy* LTR retroelements has been reported for three of the five species analysed^20^. In addition, the large-scale analysis of the Mexican axolotl genome has evidenced a recent burst of LTR retroelement expansion also for Ambystomatidae^1^.

Although the transposition of TEs can be source of genetic diversity and regulatory innovations, such events are also known to have deleterious effects, which may lead to a decrease of host fitness. To counteract the steady threat posed by TE transposition and novel insertions, diverse lineages have evolved a number of different protective mechanisms. The Ago proteins interact with small interfering RNAs (siRNAs) and microRNAs (miRNAs) to degrade the mRNAs, to repress translation, and to form heterochromatin^21^. These small RNAs are produced through the activity of DROSHA and DICER proteins. The former acts in the nucleus, loading double-stranded RNA substrates, helped by the DiGeorge Syndrome Critical Region Gene 8 (DGCR8), and modifying them to produce miRNA precursors. DICER cleaves both these molecules and endogenous or exogenous siRNAs in the cytoplasm. In germline cells, PIWI proteins are guided by small non-coding RNAs, called piRNAs (PIWI-interacting RNAs), to transcriptionally and post-transcriptionally silence transposons through base complementarity. piRNAs are extremely heterogeneous since they derive from transposons inserted in piRNA clusters, genomic regions transcribed as long single-stranded precursor RNA molecules, later processed into mature piRNAs. The transcription of these genomic regions seems to be activated by the deposition of histone 3 lysine 9 trimethylation (H3K9me3) by the protein SETDB1^22^. Subsequently the piRNA precursor transcripts are shuttled by the HMG protein Maelstrom from the nucleus to the cytoplasm, where they are cleaved by the PLD6 nuclease to form the primary piRNAs and by the HMG protein Maelstrom to form primary and secondary piRNAs^23,24^.

Additional strategies can be used to repress mobile elements. Krüppel-associated box domain zinc finger proteins (KRAB-ZFPs), the largest family of transcriptional regulators in higher vertebrates, are notably involved in the silencing of TEs, including endogenous retroviruses, LINE and SINE retroelements^25^. KRAB-ZFPs contain the KRAB domain and bind DNA through a C-terminal array of zinc finger motifs, and the Tripartite Motif Containing 28 (TRIM28) protein through their N-terminal KRAB domain. TRIM28, known also as KRAB-associated protein 1 (KAP1), serves as a scaffold for binding the heterochromatin protein 1 (HP1) and the DNA Methyltransferases (DNMT1 and DNMT3A), which allow the deposition of transcriptional repressive marks. Moreover, TRIM28 triggers the formation of heterochromatin by also recruiting the histone methyltransferase SETDB1, which adds the H3K9me3 mark, and the nucleosome remodelling and deacetylase (NuRD) complex^26,27^. This multiprotein chromatin remodelling complex contains the histone deacetylases 1 and 2 (HDAC1 and HDAC2), the chromatin helicase DNA binding protein 4 (CHD4), the Retinoblastoma-binding protein 4 and 7 (Rbbp4 and Rbbp7), either the zinc-finger proteins GATA Zinc Finger Domain Containing 2A or 2B (GATAD2a and GATAD2b), two Metastasis-associated Proteins (MTA1, MTA2, and/or MTA3), and the Methyl-CpG Binding Domain Protein 2 or 3 (MBD2 or MBD3)^28–30^. Although this mechanism was initially presumed to only act during early development, an increasing number of papers suggests that it may be also functional in adult tissues^31–34^.

The host TE silencing mechanisms evolve to counteract the rapidly mutating TEs and invasion of new TE families in the germline cells according to the “arm race” model. In mammals, the expansion of KRAB-ZFPs is positively correlated with the number of endogenous LTR elements^35^. The lack of silencing mechanisms efficiency might lead to an increase in genome size due to TE proliferation. In the lungfish giant genome, Meyer and colleagues (2021)^36^ have hypothesized that transposon silencing machinery did not adapt to reduce the TE expansion. In the strawberry poison frog, it has been proposed that its large genome might be the result of TE proliferation not suppressed by Piwi proteins in female gonad^37^.

Moreover, in the genomes expanded because of TEs DNA methylation seems to be essential for the long-term accommodation of these mobile elements in the host genome^38^.

In light of these observations, we analysed the activity of transposable elements and 33 genes encoding proteins involved in the TE host silencing mechanisms in the transcriptomes of the Chinese fire-bellied newt *Cynops orientalis* (David, 1873), a species with a genome about 12-fold larger than the human genome. Moreover, comparisons were performed with transcriptomic data obtained from tissues of the coelacanth *Latimeria menadoensis*^39^ and the lungfish *Protopterus annectens*^40^, two species characterized by significant differences in the activity of TEs and in genome size^2,41^. The data obtained showed a higher TE activity in the newt, with a major impact of non-LTR retroelements. The transcriptional activity of genes involved in TE silencing mechanisms highlighted that, although these mechanisms are active in adults, differences in TE activity evidenced between the species compared in this study may be due to the presence of old and inactive copies. In addition, to increase knowledge about the TE silencing mechanisms in amphibians, phylogenetic analyses were performed on *PRDM9* and *TRIM28*, two key genes of the NuRD complex. The former gene (also named *Meisetz*) is considered the ancestor of the KRAB domain^42^ and its absence in amphibians, crocodiles, and birds led to support its lost in these lineages^43^. The latter gene belongs to the *TRIM* family, highly diversified in vertebrates^44^, and acts as platform in the recruitment of NuRD complex proteins. Our results revealed the presence of *PRDM9* in Gymnophiona, the sister group of Anura and Caudata, and the presence of *TRIM28* in the three amphibian clades (Gymnophiona, Urodela, Anura).

## Results

### Transposable element activity in gonadal tissues in *C. orientalis*

**Figure 1:**
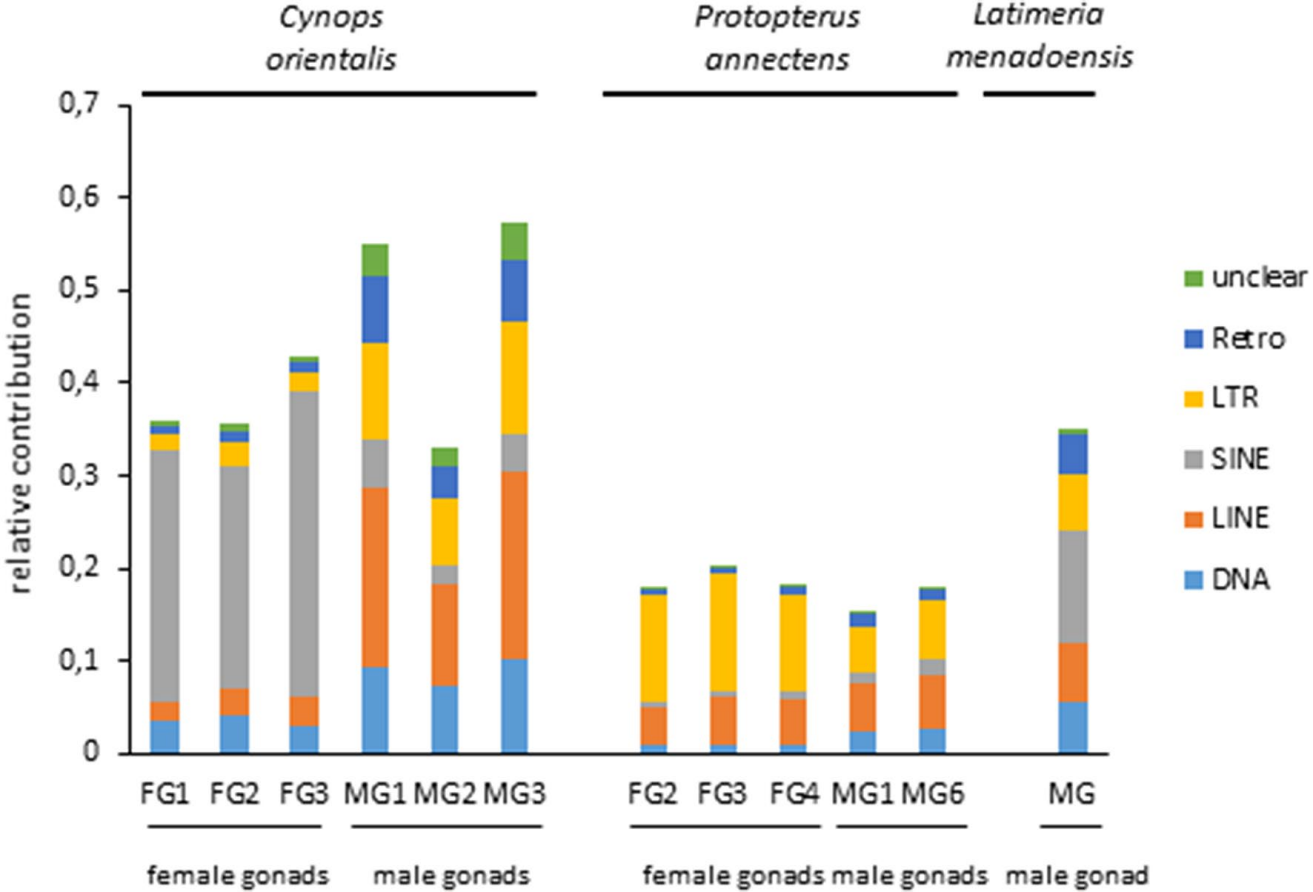
Relative contribution of transposon transcriptional activity in the transcriptomes obtained from gonadal tissues of *Cynops orientalis, Protopterus annectens,* and *Latimeria menadoensis*. *FG* female gonad, *MG* male gonad.

The transcriptional activity of TEs was investigated in the ovary and testis transcriptomes of three female and three male specimens of *C. orientalis* (Fig. 1). In the females, the analysis showed a higher relative contribution of SINE retroelements, in contrast with males, where the strongest impact was due to LINE retroelements. However, the transcriptional contribution of other TE types was not negligible in testes. The comparison between TE activity in the gonadal tissues of *P. annectens*, *L. menadoensis*, and *C. orientalis* revealed that the lowest activity was found in lungfish (Fig. 1). Moreover, the highest activity of LTR retroelements was observed in *P. annectens*, while SINE retroelement activity prevailed in *L. menadoensis* (for statistical support see Supplementary Table S1).

### Identification and transcriptional activity of genes involved in TE silencing mechanisms

**Figure 2:**
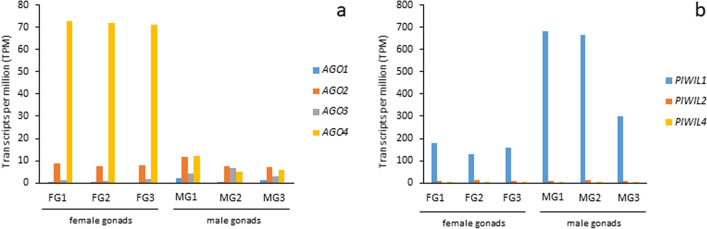
Expression levels of *AGO* genes. **(a)** Expression values of *Ago* genes detected in *Cynops orientalis* transcriptomes obtained from the female and male gonadal tissues. **(b)** Expression values of *Piwi* genes detected in *C. orientalis* transcriptomes obtained from the female and male gonadal tissues. *FG* female gonad, *MG* male gonad.

Four transcripts (*AGO1*, *AGO2*, *AGO3*, and *AGO4*) related to the *Ago* subfamily and three transcripts (*PIWIL1*, *PIWIL2*, and *PIWIL4*) related to the *Piwi* subfamily were retrieved in the female and male gonadal transcriptomes of *C. orientalis* (Supplementary Table S2). All transcripts included a complete CDS with the exception of *AGO1,* which was truncated at the 5’ and 3’ ends. As in vertebrates other than eutherian mammals, no sequence ascribable to the *PIWIL3* gene was identified in the newt transcriptomes. The evaluation of the expression levels of *Ago* genes in female gonads of *C. orientalis* (Fig. 2a) showed a very high activity of *AGO4*, followed by *AGO2*. The expression of the other two *Ago* genes was lower. Although *AGO4* gene was expressed in newt testis, we evidenced an expression level much lower than in female gonads (for statistical support see Supplementary Table S1). The comparison with the results previously obtained in lungfish and coelacanth revealed a similar pattern of expression for *AGO2* and *AGO4* in the female gonads of newt and lungfish, with lower expression levels in *P. annectens* (Supplementary Fig. S1).

**Figure 3:**
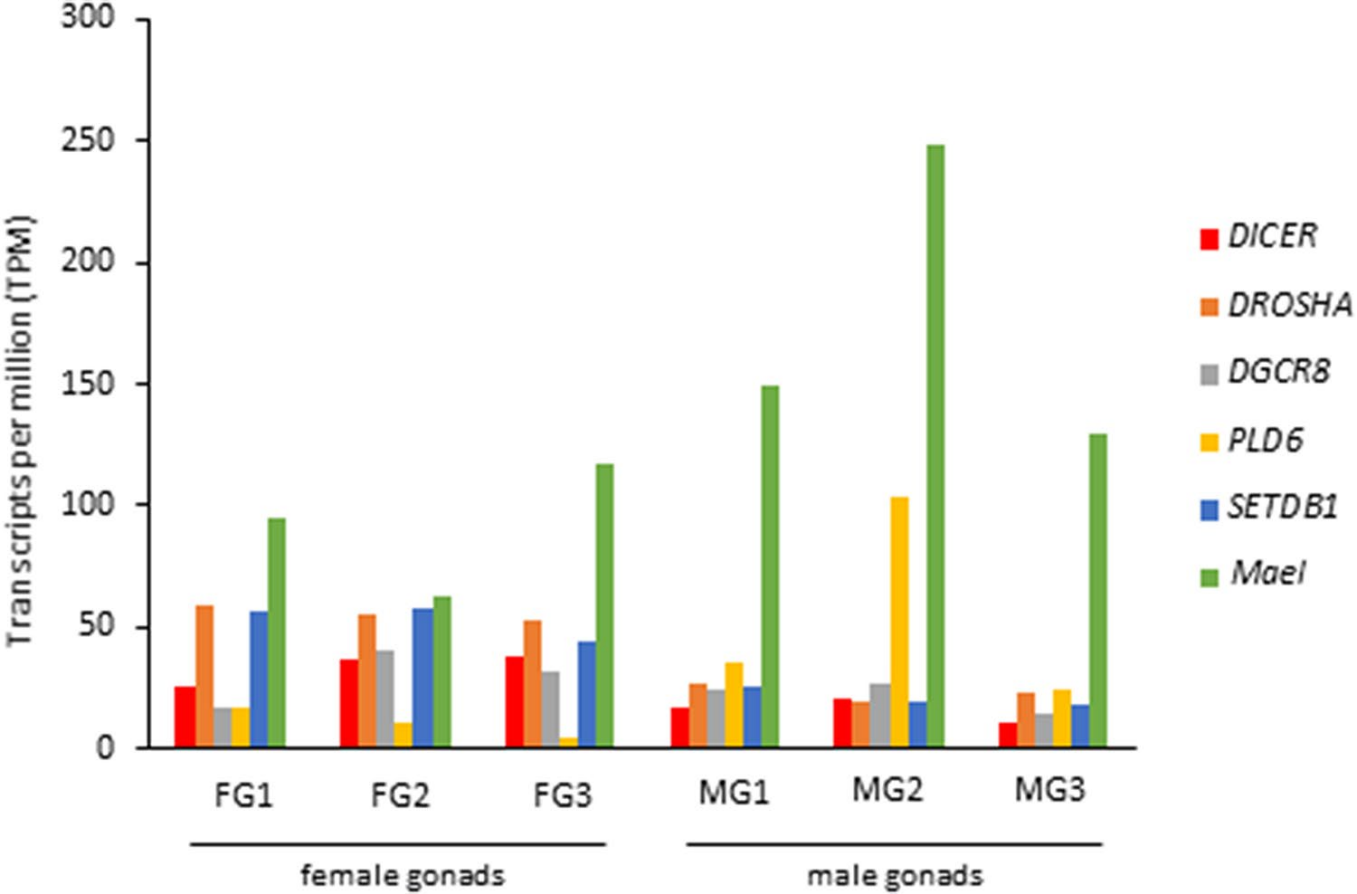
Expression levels of genes involved in small RNA biogenesis. Expression values of genes encoding proteins involved in small RNA production detected in *Cynops orientalis* transcriptomes obtained from the female and male gonadal tissues. *FG* female gonad, *MG* male gonad.

**Figure 4:**
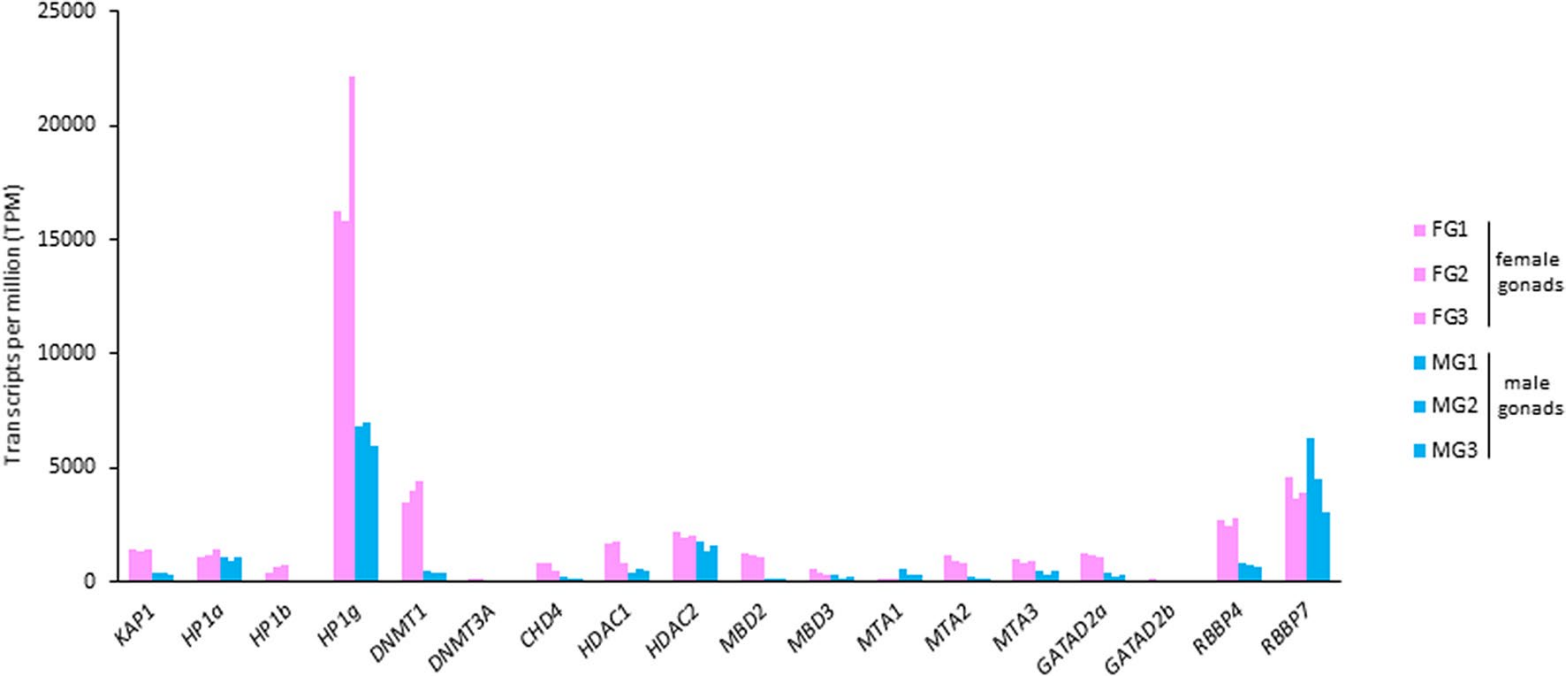
Expression levels of genes involved in heterochromatin formation and genes encoding proteins of the NuRD complex. Expression values of 18 genes investigated in *Cynops orientalis* transcriptomes obtained from the female and male gonadal tissues. *FG* female gonad, *MG* male gonad.

Concerning the *Piwi* genes, investigation of their expression levels revealed a high level of expression of *PIWIL1* in both ovary and testis transcriptomes (Fig. 2b). The transcriptional activity of *PIWIL1* in *C. orientalis* female gonads was comparable to that observed in lungfish ovaries (Supplementary Fig. S2). We also investigated the presence of expressed transcripts orthologous with genes involved in miRNA and siRNA biogenesis pathways in the transcriptomes of *C. orientalis*. Three transcripts containing the complete CDS of *DICER*, *DROSHA*, and *DGCR8* (Supplementary Table S2) were found to be expressed in all the samples analysed (Fig. 3). Similarly, three transcripts containing a complete CDS and involved in piRNA production were also expressed in all tissues, with *Mael* in particular showing high expression values in both ovary and testis (Fig. 3). Although all the six genes involved in small RNA biogenesis were actively transcribed in the fire-bellied newt, their expression levels were significantly lower than those observed in lungfish and coelacanth (Supplementary Fig. S3). Moreover, transcripts containing complete CDS were identified for *TRIM28*, the three *HP1* (*HP1a*, *HP1b*, and *HP1g*) and the two *DNMT* genes (*DNMT1* and *DNMT3A*) as well as for 12 genes encoding proteins of NuRD complex (Supplementary Table S2). The analysis of the transcriptional activity showed a high level of expression of *HP1g* and between the two methyltransferases analysed the *DNMT1* was more active in gonads (Fig. 4). Overall, the genes encoding proteins that are part of the NuRD complex were expressed at considerably high levels, with the lone exception of *GATAD2b* (Fig. 4). Moreover, these genes were also expressed in *P. annectens* and in *L. menadoensis*, but at lower levels than those observed in the newt (Supplementary Fig. S4). The transcriptional activity of these genes in the liver of the three species was found to be lower than in the gonadal tissues (Supplementary Fig. S4).

### Phylogenetic analyses and evolutionary history of *PRDM9* and *TRIM28*

**Figure 5:**
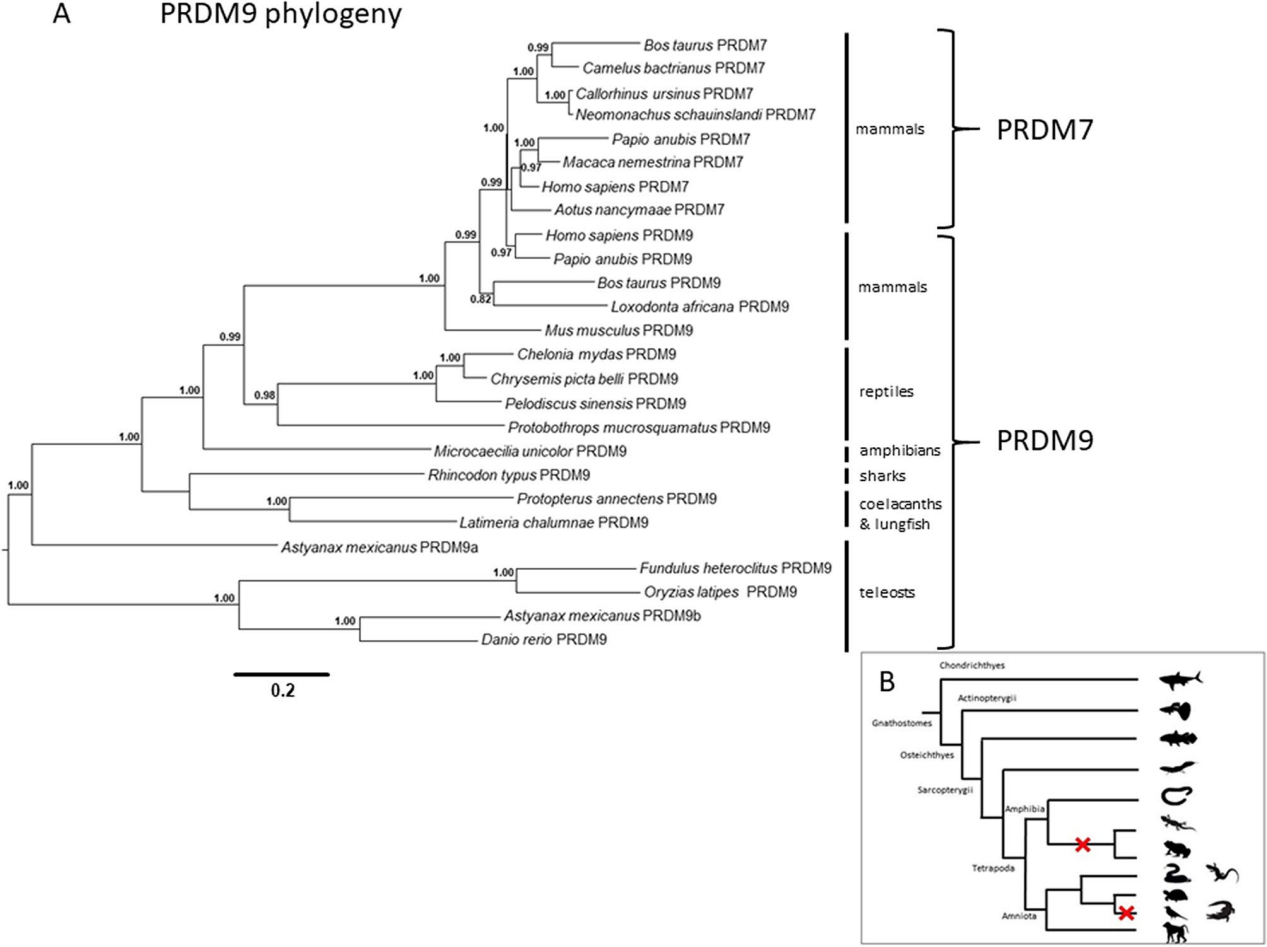
PRDM9 phylogeny. **(A)** Phylogenetic analysis of PRDM9 performed through Bayesian inference. The two curly brackets group PRDM7 and PRDM9 sequences. Vertical bars indicate to which animal group the sequences analysed belong to. **(B)** Schematic representation of *PRDM9* evolutionary history in gnathostomes. Red cross indicates gene loss.

No sequence orthologous to *PRDM9* could be identified in the available transcriptomes. However, investigation of the genome of the caecilian *Microcaecilia unicolor* revealed a *bona fide PRDM9* sequence. The orthology assessment of this sequence was confirmed by phylogenetic analysis (Fig. 5A), which also demonstrated the loss of this gene in Anura and Caudata (Fig. 5B). The protein domains of PRDM9 and PRDM7 sequences considered in the phylogenetic analysis were investigated, highlighting the presence of a KRAB A box in basal sarcopterygians and in *M. unicolor* (Supplementary Fig. S5). PRDM7 sequences were present only in mammals.

**Figure 6:**
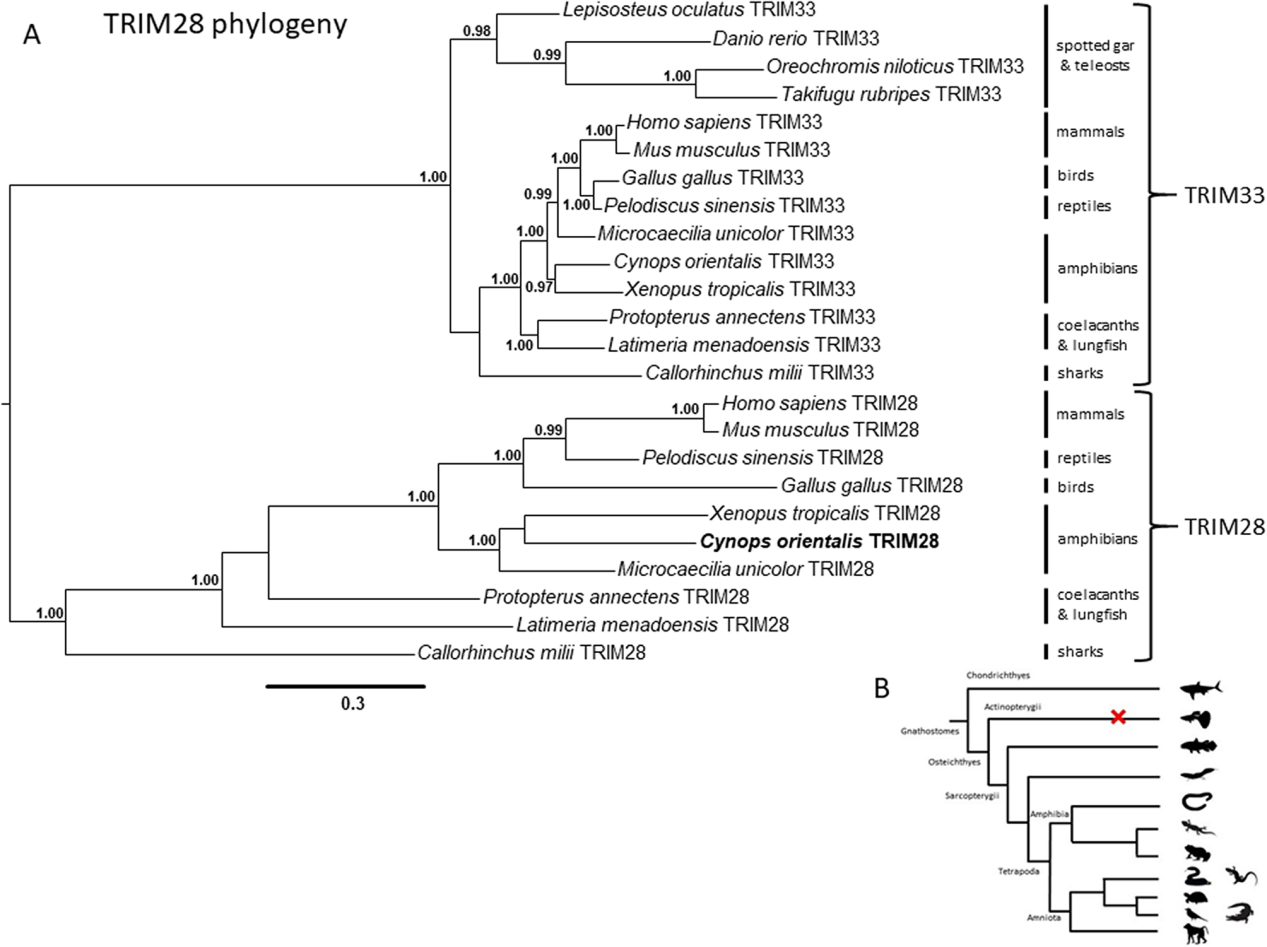
TRIM28 phylogeny. **(A)** Phylogenetic analysis of TRIM28 performed through Bayesian inference. The two curly brackets group TRIM33 and TRIM28 sequences. Vertical bars indicate to which animal group the sequences analysed belong to. **(B)** Schematic representation of *TRIM28* evolutionary history in gnathostomes. Red cross indicates gene loss. *Cynops orientalis* TRIM28 is in bold.

Phylogenetic analysis also confirmed the presence of *TRIM28* in *C. orientalis* (Fig. 6A), as well as in *Xenopus tropicalis* and *M. unicolor*, which supports the presence of this gene in all three amphibian lineages. Moreover, a TRIM28 sequence was also identified in the elephant shark *Callorhinchus milii* (Fig. 6B).

## Discussion

Salamanders and lungfish display the largest genomes among vertebrates^15^. This characteristic was specifically acquired along the Caudata lineage, since the ancestor of modern Lissamphibia (salamanders and frogs) likely had a nuclear DNA content of 5–10 pg^45,46^. In extant salamanders, genome expansion was attributed to the presence of long introns, slow rates of DNA loss, and high density of TEs, in particular of LTR retroelements^1,18–20^. In axolotl, where repetitive sequences account for 65.6% of the genome assembly size, the estimated relative age of the LTR retroelements permitted identification of a long period of activity followed by a recent burst of expansion^1^. The study of the amphibian genome size variation is even more interesting if we consider that organisms with small genomes, similar to those of birds, are present in this lineage. The recent genome sequencing of the ornate burrowing frog *Platyplectrum ornatum* has shown that reduced genome size is correlated with shorter intron lengths, low amount of TEs, and an increased expression of Piwi pathway genes^47^.

The evolutionary processes underlying the accumulation and persistence of high levels of repetitive sequences in these organisms are not completely understood.

### TE activity in gonadal transcriptomes of *C. orientalis*

The relative contribution of TE activity in the ovary and testis transcriptomes of three *C. orientalis* individuals of each sex suggested a major impact of non-LTR retroelements (Fig. 1). Nevertheless, we need to point out that, in absence of a complete genome sequence for this species, we cannot establish whether this observation simply reflects a higher transcriptional activity or if it indicates a parallel genome-wide expansion of this TE class. If the high amount of LTR retroelements found in common among salamander genomes is also shared by the fire-bellied newt, this finding might suggest either a lower activity of LTR elements in gonads or the presence of old/degenerate copies. Moreover, in female gonads, SINE strongly prevailed as the most active non-LTR retroelements. Although LINE retroelements were the most active elements in male gonads, all TE types showed a considerable transcriptional contribution. TEs are well-known potential regulators of gene networks that may influence the expression of neighbouring genes by hitchhiking promoters, transcription factor binding sites, insulators, splicing sites or epigenetic modifications^21^. The different pattern of TE activity observed in gonads could be due to their involvement in the regulatory toolkit of ovaries and testes in *C. orientalis*, which would be in line with the different transcriptomic expression profiles obtained in these tissues in our previous work^44^. Moreover, inter-sex variations in the activity of TEs in gonads have been also previously described in other vertebrates^49,50^.

### The impact of TEs and mechanisms for transposition silencing in giant genomes

The low TE activity values observed in the lungfish *P. annectens*, compared with *C. orientalis*, is particularly intriguing since the two species share a comparable genome size. A possible explanation for this discrepancy might be sought in the different expression of genes involved in TE silencing mechanisms in the two species. It is noteworthy that the *Ago* and *Piwi* gene activity was found to be significantly higher in the newt. On the other hand, the genes involved in heterochromatin formation and those encoding proteins of the NuRD were actively transcribed in both species. In light of these observations, the lower TE activity in *P. annectens* might be consistent with the hypothesis that its genome is mainly made up of nonactive mobile elements^23,40,51,52^. Indeed, the genomic expansion in lungfish lineage occurred independently^36,45^ and earlier than that of salamander lineage. In lungfish, the increasing in genome size can be dated in the Carboniferous (360–320 Mya) while in amphibians the expansion started in the Permian (280 Mya) up to the Middle Jurassic (208 Mya)^46,53,54^. The recent genome sequencing of the West African lungfish has provided further support to the hypothesis of an ancient burst of transposition followed by a long period of degeneration creating a “cemetery of TEs”^2,55^. Moreover, the high number of KRAB-ZFPs identified in the genome of the African lungfish has also suggested that this species evolved a higher ability to repress TEs^2^. Taking into account the genome size and the high transcriptional levels of the genes here investigated, we hypothesize that the TE repression system may have also been enhanced in *Cynops*. By contrast, coelacanths did not evolve a strong TE repression toolkit due to the low number of TEs in their genome, about 12-fold lower than those of the fire bellied newt and lungfish. This idea is consistent with the expression levels of TEs and genes encoding proteins involved in heterochromatin formation and silencing in this species. Overall, the activity of TEs can increase due to environmental stressors such as temperature^22^. In particular, the beginning of Carboniferous and the transition from Permian to Triassic were characterized by severe environmental changes^56^ that might have provoked a reduction of the efficiency of silencing mechanisms in gonads, leading to a mobilization of TEs and thus to an increase of genome size in lungfish and newts^17^.

The transcriptional activity of *TRIM28*, *HP1*, *DNMTs*, and NuRD complex genes in adults supports the evidence that these silencing mechanisms are not only active in early developmental stages^31–34^. Moreover, the levels of expression of these genes were quite high in gonads compared to the somatic hepatic tissue. This finding might be correlated with the need to repress TE activity and to preserve genome integrity in gonads. The NuRD complex is involved in endogenous retrovirus silencing^33^ and consequently the high expression levels of its genes in *C. orientalis* suggested a high prevalence of these elements in the newt genome. This hypothesis is in line with the results that emerged from the complete genome sequencing of axolotl, which revealed that LTR retroelements and endogenous retroviruses were the most abundant classes of repetitive sequences^1^. Moreover, the high transcriptional activity of the *DNMT1* methyltransferase suggested this gene as the main candidate regulator of TE methylation in *C. orientalis*. This mechanism has been proposed as essential for the long-term accommodation of TEs in the host genome^38^.

### TE silencing mechanisms evolved in amphibians as in other tetrapods

Phylogenetic analyses were focused on *PRDM9* and *TRIM28*, two genes of interest to increase our knowledge about the evolution of the KRAB-ZFPs and NuRD complex, involved in TE silencing mechanisms in vertebrates. It is known that the KRAB domain contained in the KRAB-ZFPs derived from *PRDM9* gene^42^. This gene shows an interesting evolutionary history since it was lost in crocodiles, birds and, among amphibians, in salamanders and frogs^43^. Our analysis confirmed the absence of this gene in *C. orientalis*. However, we identified *PRDM9* in the caecilian *M. unicolor,* demonstrating that its loss in the amphibian lineage might have occurred in the common ancestor of frogs and salamanders, after the split from the Gymnophiona (caecilian) lineage. On the other hand, TRIM28 was found in all three amphibian lineages. Moreover, the identification of a *TRIM28* sequence in the elephant shark *C. milii* suggests that this gene was already present in the common ancestor of gnathostomes and consequently hints that the absence of this gene in Actinopterygii was due to a secondary loss. The presence of *PRDM9* and *TRIM28* genes suggested that the TE silencing mechanisms evolved in amphibians as in other tetrapods.

## Conclusions

The findings here obtained highlighted a major impact of non-LTR retroelements and a greater total TE activity in the newt *C. orientalis* compared to the lungfish *Protopterus annectens*, another organism characterized by a giant genome. Therefore, the activity and accumulation of TEs likely played a key role in the genomic gigantism in urodeles. In particular, the difference in TE activity between the newt *Cynops* and the lungfish *Protopterus* might be due to the presence of younger and active copies in newt.

The transcriptional activity of target genes encoding proteins involved in the TE host silencing mechanisms confirmed that the NuRD complex was active also in adults and that their expression was high in gonads to preserve genome integrity. Further comparative studies that may include species characterized by smaller genomes will provide new insights on the evolutionary “arms-race” between TEs proliferation and silencing mechanisms acting in host genome.

Finally, our phylogenetic analyses showed that *PRDM9* was present in the common ancestor of amphibians and *TRIM28* was present in the common ancestor of gnathostomes. Therefore, these findings allowed to increase knowledge about the evolution of these two key genes of NuRD complex silencing mechanism in vertebrates.

## Material and methods

### Identification of transposable elements and estimate of transcriptional activity

The identification of expressed transposable elements was carried out using the methodology described in a previous work^40^. Briefly, the de novo assembled transcriptome of *C. orientalis*^48^ was first masked with RepeatMasker (http://www.repeatmasker.org/cgi-bin/WEBRepeatMasker) against the Dfam^57^ database, and then scanned with RepeatScout^58^ to identify novel active repeated elements. Following a false positive decontamination step aimed at removing protein-coding sequences not ascribable to TE-associated conserved domains, the elements were classified with TEClass^59^ into the following categories: DNA transposons, LTR retroelements, LINEs, SINEs, retroelements (whenever a retroelement could not be discriminated with certainty as a LTR or non –LTR retroelements) or unclear. The likely collapse of nearly identical TEs belonging to the same family, as well as the frequent fragmentation of transcripts containing repeats, typical of transcriptome de novo assembly approaches^60^, prevented the classification of the identified TEs on a finer scale.

The cumulative activity of each class of TEs was calculated in *C. orientalis*, *P. annectens*, and *L. menadoensis* available gonadal tissue samples, as their relative contribution to the total transcriptional effort, i.e. by calculating the fraction of reads mapped to the elements included in the repeat library, relative to the total number of reads mapped to the reference transcriptome assemblies in each sample. This task was carried out with the proprietary *map reads to reference* tool included in the CLC genomics Workbench v.20 (Qiagen, Hilden, Germany), using the following mapping parameters: length fraction = 0.75, similarity fraction = 0.98. TE counts for *C. orientalis* were listed in the Supplementary Table S3. The statistical significance between the relative abundance of any TE class between male and female gonads was evaluated with an unpaired t-test (Supplementary Table S1).

### Characterization and expression of genes involved in TE silencing mechanisms

The de novo transcriptome assembly obtained from the ovary and testis collected from three *C. orientalis* specimens of each sex (FG1/FG2/FG3 and MG1/MG2/MG3, respectively) was used as a reference database for sequence homology searches^48^ with tBLASTn^61^. In detail, the search involved transcripts orthologous to members of the *Ago* (*AGO1*, *AGO2*, *AGO3*, *AGO4*) and *Piwi* (*PIWIL1*, *PIWIL2*, *PIWL4*) subfamilies, as well as those involved in small RNA biogenesis (*DICER*, *DROSHA*, *DGCR8*, *PLD6*, *SETDB1*, *Mael*). In addition, the search was extended to *P. annectens*^40^ and *L. menadoensis*^39^ for *PRDM9*, *TRIM28*, *HP1a*, *HP1b*, *HP1g*, *DNMT1* and *DNMT3A*, and for the transcripts encoding proteins of the NuRD complex (*CHD4*, *HDAC1*, *HDAC2*, *MBD2*, *MBD3*, *MTA1*, *MTA2*, *MTA3*, *GATAD2a*, *GATAD2b*, *RBBP4*, *RBBP7*). All transcripts were translated using Sequence Translation (https://www.ebi.ac.uk/Tools/st/), allowing the identification of UTR and CDS regions (Supplementary Table S2). The sequences were deposited in GenBank under the accession numbers provided in the Supplementary Table S2. The presence of conserved protein domains in PRDM9 and its paralog PRDM7 was inferred with CD-Search^62^.

The expression values of genes of interest are reported as transcripts per million (TPM) to allow comparisons both within and between tissues^63^ (Supplementary Table S3). These values were further adjusted following the procedure described in Biscotti et al. 2016^48^ to ensure full compatibility with data previously published for other species. Briefly, to take into account possible distortions of TPM calculations linked with the different structure of the transcriptomes, gene expression values were computed by using a scaling factor, based on the cumulative expression of 2111 broadly expressed verified single-copy orthologs shared by all the species taken into account. The expression levels of such genes were calculated with the CLC Genomics Workbench v.20, using the following parameters: length fraction = 0.75, similarity fraction = 0.98. Read counts obtained from the three biological replicates of male and female gonads, normalized by quantile, were used to perform a statistical analysis of differential gene expression, carried out with a Baggerlys’ test^64^. The presence of the genes of interest among the subset of those differentially expressed between the gonads of the two sexes was ascertained based on a Bonferroni-corrected p-value < 0.05 (Supplementary Table S1).

### Phylogenetic analyses

Phylogenetic analyses were performed to assess the orthology and the evolutionary history of genes of interest. Orthologous Similar sequences were retrieved from GenBank (http://www.ncbi.nlm.nih.gov/) or ENSEMBL (http://www.ensembl.org/index.html) and used in the phylogenetic analyses (for accession numbers see Supplementary Table S4). Multiple alignments of the amino acid sequences were obtained with Clustal Omega (https://www.ebi.ac.uk/Tools/msa/clustalo/) using default parameters. PRDM9 and TRIM28 phylogenetic analyses with midpoint rooting were performed by Bayesian inference using MrBayes (version 3.2)^65^ and 1,000,000 generations were run (the burn-in was set to 2,500). The Jones amino acid model^66^ was identified by the MrBayes program with a potential scale reduction factor (PSRF) of 1.00. Stationarity defined as the condition where the average standard deviation of split frequencies was of 0.0019 for PRDM9 phylogeny and of 0.0051 for TRIM28 phylogeny.

## Supplementary Information

Supplementary Table S1.

Supplementary Table S2.

Supplementary Table S3.

Supplementary Table S4.

Supplementary Figure S1.

Supplementary Figure S2.

Supplementary Figure S3.

Supplementary Figure S4.

Supplementary Figure S5.

Supplementary Legends.
