# Supplementary figures and images for: Investigation of the activity of transposable elements and genes involved in their silencing in the newt Cynops orientalis, a species with a giant genome
Source: Sci Rep. 2021 Jul 20;11:14743. doi: 10.1038/s41598-021-94193-6 (PMC8292531; doi:10.1038/s41598-021-94193-6)

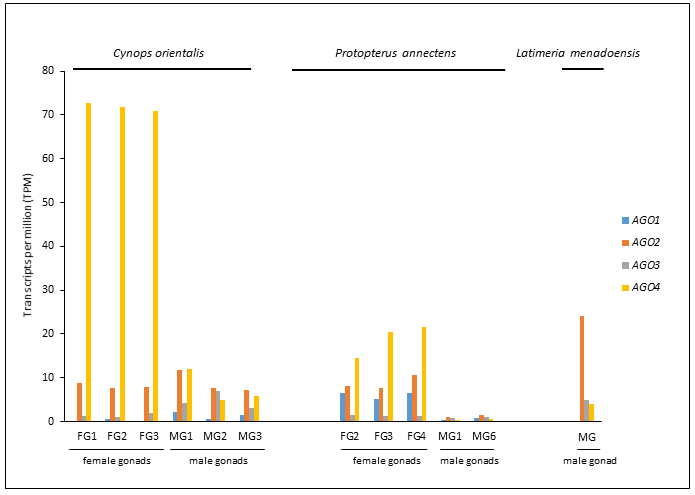

Supplement: Supplementary file 5 — Supplementary Figure S1. [file 41598_2021_94193_MOESM5_ESM.tif]

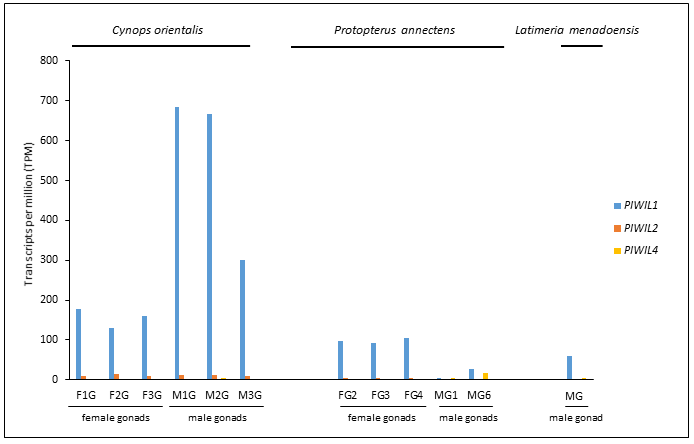

Supplement: Supplementary file 6 — Supplementary Figure S2. [file 41598_2021_94193_MOESM6_ESM.tif]

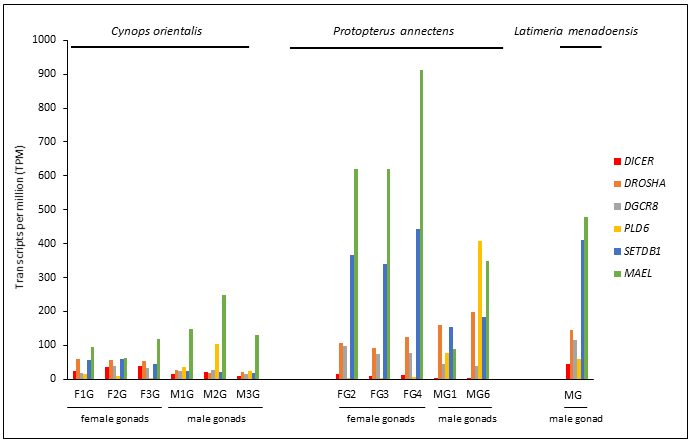

Supplement: Supplementary file 7 — Supplementary Figure S3. [file 41598_2021_94193_MOESM7_ESM.tif]

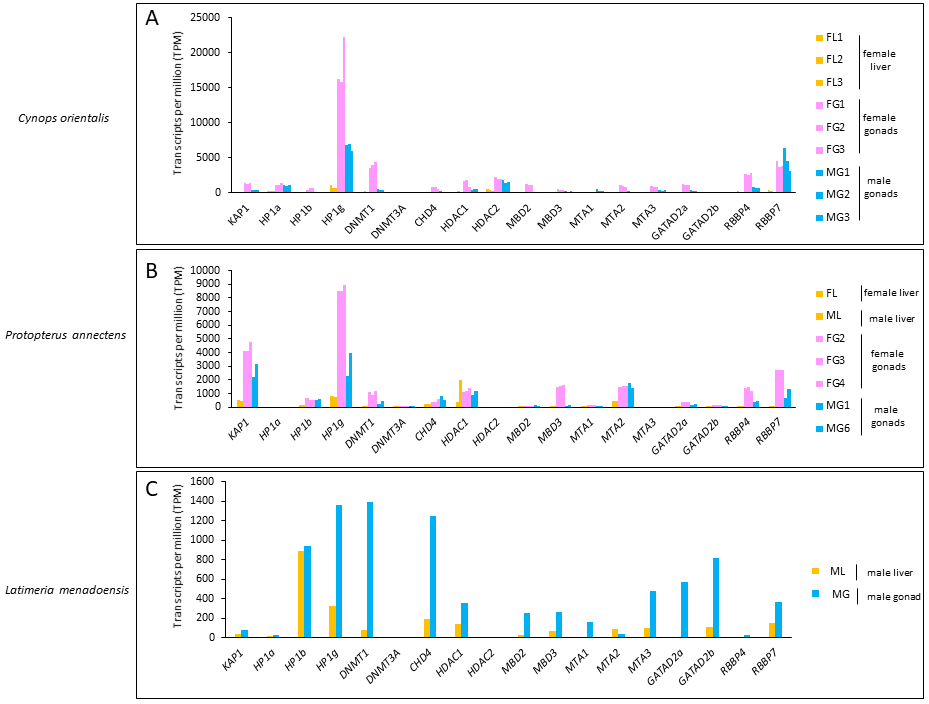

Supplement: Supplementary file 8 — Supplementary Figure S4. [file 41598_2021_94193_MOESM8_ESM.tif]

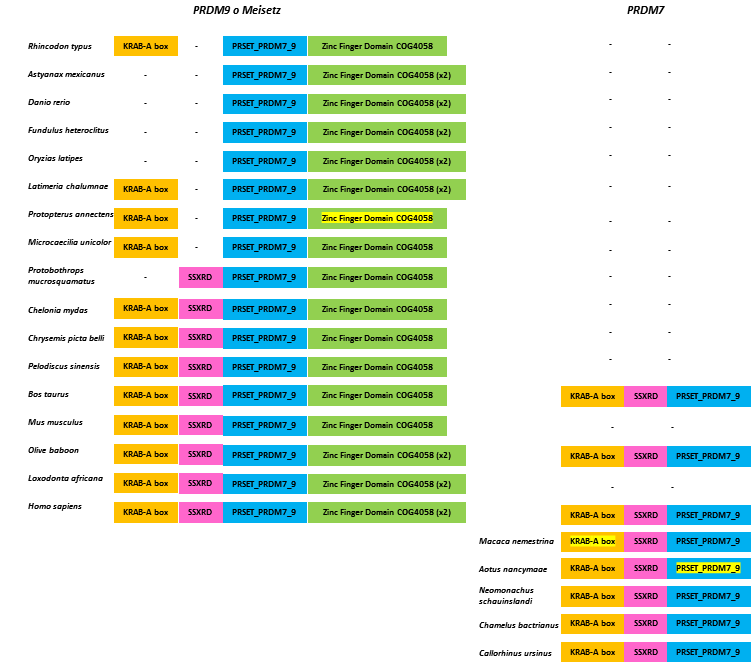

Supplement: Supplementary file 9 — Supplementary Figure S5. [file 41598_2021_94193_MOESM9_ESM.tif]
