# Supplementary material for: Investigation of the activity of transposable elements and genes involved in their silencing in the newt Cynops orientalis, a species with a giant genome
Source: Sci Rep. 2021 Jul 20;11:14743. doi: 10.1038/s41598-021-94193-6 (PMC8292531; doi:10.1038/s41598-021-94193-6)
Supplement: Supplementary file 10 — Supplementary Legends. [file 41598_2021_94193_MOESM10_ESM.pdf]

Supporting Information

Investigation of the activity of transposable elements and genes involved in their silencing in the newt *Cynops orientalis*, a species with a giant genome.

Federica Carducci[1], Elisa Carotti[1], Marco Gerdol[2], Samuele Greco[2], Adriana Canapa[1], Marco Barucca[1]*, Maria Assunta Biscotti[1]

Supplementary Figure S1. Expression levels of *Ago* genes in the transcriptomes obtained from gonadal tissues of *Cynops orientalis, Protopterus annectens,* and *Latimeria menadoensis*. FG: female gonad; MG: male gonad.

Supplementary Figure S2. Expression levels of *Piwi* genes in the transcriptomes obtained from gonadal tissues of *Cynops orientalis, Protopterus annectens,* and *Latimeria menadoensis*. FG: female gonad; MG: male gonad.

Supplementary Figure S3. Expression levels of genes involved in small RNA biogenesis in the transcriptomes obtained from gonadal tissues of *Cynops orientalis, Protopterus annectens,* and *Latimeria menadoensis*. FG: female gonad; MG: male gonad.

Supplementary Figure S4. Expression levels of genes involved in heterochromatin formation and genes encoding proteins of the NuRD complex. A) Expression values of genes coding for proteins involved in heterochromatin formation and genes of the nucleosome remodelling and deacetylase (NuRD) complex detected in *Cynops orientalis* transcriptomes obtained from female liver, ovary, and testis. B) Expression values of genes coding for proteins involved in heterochromatin formation and genes of the nucleosome remodelling and deacetylase (NuRD) complex detected in *Protopterus annectens* transcriptomes obtained from liver and gonadal tissues of female and male specimens. C) Expression values of genes coding for proteins involved in heterochromatin formation and genes of the nucleosome remodelling and deacetylase (NuRD) complex detected in *Latimeria menadoensis* transcriptomes obtained from male liver and testis. FL: female liver; ML: male liver; FG: female gonad; MG: male gonad.

Supplementary Figure S5. Protein domains of PRDM9 and its paralog PRDM7 inferred through Conserved Domain Database. Not complete protein domain identification due to partial sequence are highlighted in yellow. Black dashes indicate the absence of specific protein domains or complete sequences.

Supplementary Table S1. Statistical analyses. Significance is reported as the p-value of an unpaired t-test for the comparison of the activity of TEs between *Cynops orientalis* male and female gonads and between *Cynops orientalis* and *Protopterus annectens*. Significance is reported as the Bonferroni-corrected p-value of a Baggerly's test for the comparison of the gene expression levels between *Cynops orientalis* male and female gonads.

Supplementary Table S2. Details of gene sequences identified in *Cynops orientalis*, *Latimeria menadoensis*, and *Protopterus annectens*.

Supplementary Table S3. Lists all the transposable elements detected in *Cynops orientalis*, together with their estimated levels of transcriptional activity in different biological samples reported as counts per million (CPM). Transcripts per million values related to silencing genes identified in *C. orientalis*.

Supplementary Table S4. Accession numbers of sequences used in phylogenetic analyses.
